# A reporter system for enriching CRISPR/Cas9 knockout cells in technically challenging settings like patient models

**DOI:** 10.1038/s41598-021-91760-9

**Published:** 2021-06-16

**Authors:** Wen-Hsin Liu, Kerstin Völse, Daniela Senft, Irmela Jeremias

**Affiliations:** 1 Research Unit Apoptosis in Hematopoietic Stem Cells, German Research Center for Environmental Health (HMGU), Helmholtz Zentrum München, Marchioninistraße 25, 81377 Munich, Germany; 2 German Cancer Consortium (DKTK), Partner Site Munich, Munich, Germany; 3 Department of Pediatrics, Dr. von Hauner Children’s Hospital, Ludwig Maximilian University, Munich, Germany

**Keywords:** Cancer models, Genetic engineering

## Abstract

CRISPR/Cas9 represents a valuable tool to determine protein function, but technical hurdles limit its use in challenging settings such as cells unable to grow in vitro like primary leukemia cells and xenografts derived thereof (PDX). To enrich CRISPR/Cas9-edited cells, we improved a dual-reporter system and cloned the genomic target sequences of the gene of interest (GOI) upstream of an out-of-frame fluorochrome which was expressed only upon successful gene editing. To reduce rounds of in vivo passaging required for PDX leukemia growth, targets of 17 GOI were cloned in a row, flanked by an improved linker, and PDX cells were lentivirally transduced for stable expression. The reporter enriched scarce, successfully gene-edited PDX cells as high as 80%. Using the reporter, we show that KO of the SRC-family kinase LYN increased the response of PDX cells of B precursor cell ALL towards Vincristine, even upon heterozygous KO, indicating haploinsufficiency. In summary, our reporter system enables enriching KO cells in technically challenging settings and extends the use of gene editing to highly patient-related model systems.

## Introduction

CRISPR/Cas9 gene-editing has emerged as a powerful tool to determine protein function, however, gene-editing is often an inefficient process where only a small subpopulation of cells is edited, especially in genes difficult to edit^1^. Selecting the scarce cell subpopulation of edited cells from the large majority of unedited cells is technically challenging, especially if intracellular proteins are studied. The inability to enrich cells with knockout (KO) often precludes the use of this technique in clinically relevant samples such as primary patient or patient-derived xenograft (PDX) cells, which are hard to transduce and reluctant to grow in vitro^1–4^.

Acute lymphoblastic leukemia (ALL) is the most common childhood cancer and relapsed or refractory disease is associated with a dismal prognosis in both, children and adults and novel treatment options are required^5^. Target identification and validation relies on clinically relevant model systems that faithfully recapitulate the heterogeneity of patient tumors^2^. While primary patient samples are readily available for most leukemias, they cannot be amplified in vitro, requiring their passaging in mice for amplification^2,3^.

Here, we aimed at generating derivative PDX models with CRISPR/Cas9-mediated knockout. Towards this aim, we advanced previously published surrogate reporter systems^1,6,7^ and studied the SRC-family kinase LYN, which mediates signals from the B-cell receptor and has been suggested to play a tumor promoting role in B-ALL^8–14^. We show that our reporter system allows to successfully enrich gene edited cells, even in model systems hard to transduce and unable to grow in vitro. We demonstrate that homo- or heterozygous loss of *LYN* increased treatment response to Vincristine (VCR) in ALL cells, indicating a yet unknown haploinsufficiency of the loss of the *LYN* gene. Our reporter system expands the use of CRISPR/Cas9-mediated gene editing to technically challenging, but clinically highly relevant model systems.

## Results and discussion

We aimed at generating a reporter system for challenging cells that are unable to grow ex vivo and hard to transduce, to enable enrichment of cells with successful gene editing.

**Figure 1:**
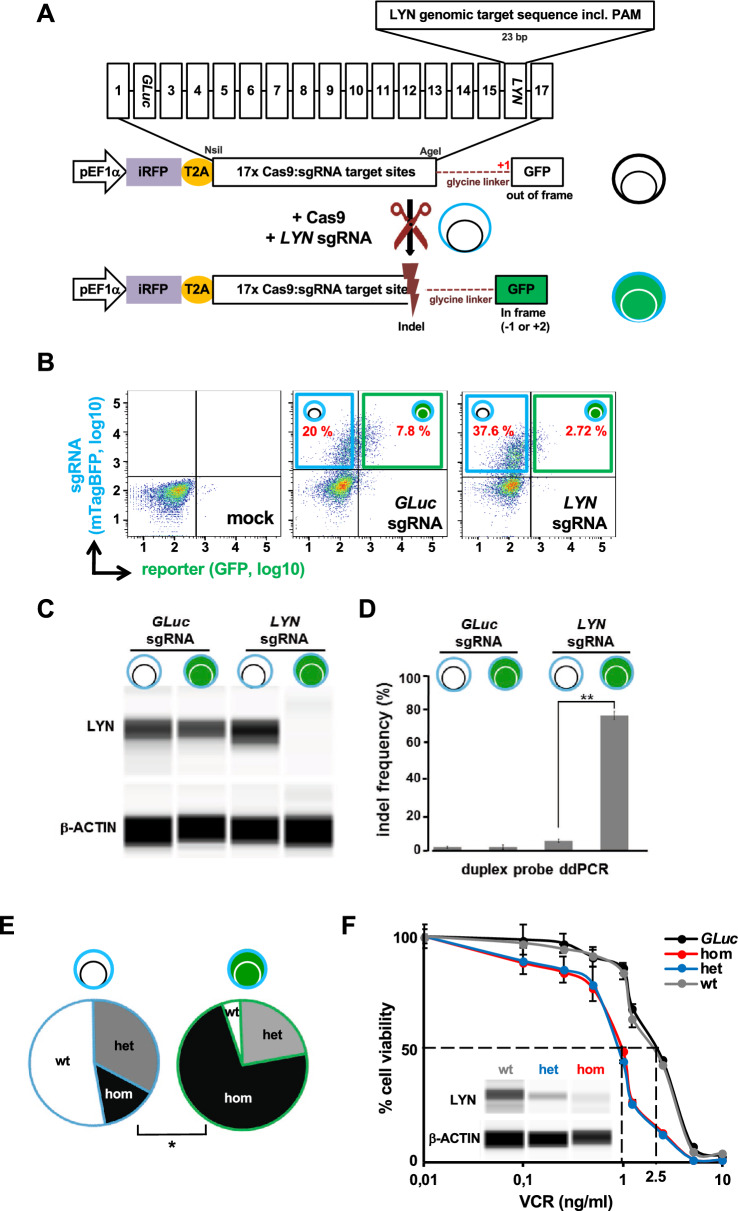
The surrogate reporter enriches CRISPR/Cas9-edited knockout (KO) cells. (**A**) *Design of the reporter;* The reporter consists of two fluorochromes, iRFP720 and GFP, both expressed from a single promoter (EF1α) and coupled by the self-cleaving peptide T2A. While iRFP720 is constitutively expressed, the GFP cassette is cloned out-of-frame by + 1 base. In the 5′ untranslated region of GFP, 17 different Cas9:sgRNA target sites were cloned in, each consisting of a copy of the genomic target sequence of the gene of interest (GOI) including its PAM sequence; the Cas9:sgRNA target sites were followed by a 48 bp glycine-rich linker (dashed line). Upon co-expression of Cas9 and sgRNA, Cas9 induced DNA double strand breaks in the specific Cas9:sgRNA target site of the reporter; non-homologous end joining (NHEJ)-mediated frame shifts (Indels), which altered the reading frame by − 1 or + 2, enabled expression of GFP. (**B**) *Gating strategy;* NALM-6 cells stably expressing Cas9 and the reporter were lentivirally transduced mock (without sgRNA construct), a *GLuc*-targeting sgRNA or a *LYN*-targeting sgRNA construct that co-expressed mTagBFP (Figure S2). 72 h after lentiviral transduction, cells were analyzed by flow cytometry for fluorochrome-marker expression. Representative histograms of *GLuc-*sgRNA-transduced, *LYN*-sgRNA-transduced and untransduced control (mock) cells are shown. mTagBFP (blue) indicated presence of the sgRNA, while co-expression of GFP (green) and mTagBFP (blue) indicated additionally reporter positive cells. (**C**) *LYN protein expression is reduced in reporter positive cells;* Automated calculated pictures of capillary immunoassay of LYN protein expression in cells sorted as in (**B**). β-ACTIN served as a loading control; one representative calculated picture out of 3 independent experiments is shown. (**D**) *Enrichment of indel frequency at the LYN locus;* Digital droplet PCR (ddPCR) was performed in cells sorted as in (**B**) to determine percentage of edited versus non-edited alleles. Mean ± SD of 3 independent experiments is shown; **p < 0.01. (**E**) *The reporter enriches KO cells* Frequency of NALM-6 single cell clones with and without heterozygous or homozygous gene deletion in the *LYN* gene; see Figure S6 for raw data and experimental details. (**F**) *LYN KO sensitizes NALM-6 cells to vincristine (VCR);* Single cell clones and *GLuc* control NALM-6 cells were incubated with the indicated concentrations of VCR for 2 days before cell viability was measured by FACS based on FCS/SCC gating. Mean ± SD of 3 independent experiments is shown. Inner panel: Automated calculated pictures of capillary immunoassay of LYN protein expression from 3 different single cell clones. β-ACTIN was used as a loading control.

Available surrogate reporter systems to enrich gene-edited cells are based on the premise that programmable nucleases that edit the surrogate reporter also edit the genomic target sequence with a high probability^1,6,7^. We chose a dual fluorochrome based system that enables selection of transfection positive and importantly, nuclease active cells (Fig. 1A). The reporter constitutively expresses iRFP-720 (iRFP) as a fluorochrome marker to enrich transduced cells using flow cytometry. The second fluorophore of the reporter, destabilized green fluorescent protein (GFP), is cloned out of frame (+ 1), so that it is only expressed if an appropriate frameshift (− 1 or + 2) is introduced upstream, by error-prone non-homologous end joining (NHEJ) following Cas9-mediated double-strand breaks (Fig. 1A and Figure S1). We decided for an "on" reporter where onset of expression of the fluorochrome indicates successful editing, because "off" reporters are hampered by the time required until expression of the reporter marker is completely vanished. While we decided for a fluorochrome marker for enrichment, any other selection strategy, like a resistance gene, can also be used. Both fluorochromes and the sequence between them is translated into protein, adding an N-terminal nonsense peptide to GFP as the T2A site was cloned 3′ of iRFP and 5′ to the Cas9:sgRNA target site to prevent putative indels in the T2A site. To detect successful editing of genes of interest (GOI), we cloned distinct Cas9:sgRNA target sites between both fluorochromes (Fig. 1A and Figure S1). Each Cas9:sgRNA target site is a copy of 20 bp of the genomic sequence of the GOI that is recognized by the specific single strand guide RNA (sgRNA), including the *Streptococcus pyogenes* Cas9 (SpCas9) 5′-NGG-3′ protospacer adjacent motif (PAM) sequence.

With the goal to use the reporter in PDX leukemia cells, we aimed to increase efficiency and reduce rounds of in vivo cell amplification. We advanced published reporters^6^ and cloned 17 Cas9:sgRNA target sites in a row; multiplexing of different Cas9:sgRNA target sites allowed generating a single transgenic PDX line with one reporter construct enabling the studies of numerous different target genes. While attractive, the accompanying challenge lies in arranging all 17 Cas9:sgRNA target sites such as to prevent premature stop codons in the entire reporter before gene editing. A stop codon existing in any of the Cas9:sgRNA target sites might prevent translation of GFP upon successful gene editing at a neighboring Cas9:sgRNA target site. As a consequence and prerequisite, each individual Cas9:sgRNA target site required absence of stop codons in at least one reading frame, while adding 1 or 2 bases between Cas9:sgRNA target sites allowed having each Cas9:sgRNA target site in the desired frame lacking stop codons (Figure S1A).

The reporter contains a non-human targeting sequence (Gaussia Luciferase, *GLuc*) at position 2 (Fig. 1A and Supplemental Table S1), which is exclusively present in the reporter and serves as a control; the remaining 16 targeting sequences can be used to select edited cells of distinct GOIs. At position 16, the reporter contains the *LYN* genomic targeting sequence (Fig. 1A and Supplemental Table S1).

To avoid undesired interference, we added a glycine linker between the most 3’ Cas9:sgRNA targeting site and GFP (Figure S2A) and introduced distancing between GFP and the nonsense peptide translated from the Cas9:sgRNA target sites. As additional advantage, the linker prevented destruction of the coding region of GFP upon large deletions by NHEJ which might increase the number of false-negative reporter cells^15,16^.

We first used a cell line system to test different aspects of the reporter and test its suitability. We chose a B-ALL cell line, NALM-6, which stably expressed human codon-optimized *S. pyogenes* Cas9 (Figure S2B). To test different lengths of linker sequences, Cas9-expressing NALM-6 cells were transduced with reporter constructs containing either no linker or an 15 or 48 bp linker (Figure S2A), and were sorted for iRFP. In a second round, cells were transduced with either an empty sgRNA (EV-sgRNA, Figure S2B) vector expressing mTagBFP as control, an ADAM17-targeting or a *LYN*-targeting sgRNA vector (expression constructs are detailed in Fig. 1A, Figure S2 and Supplemental Table S1). Compared to cells that contained the reporter without linker, the short 15 bp linker already increased marker expression within the fraction of sgRNA-expressing cells. The 48 bp linker was even more potent and enriched *ADAM17*-sgRNA or *LYN*-sgRNA and GFP double-positive cells to more than 55% and 24%, respectively (Figure S2C); false-positive rate was negligible, with only 0.77% GFP-positive cells in the EV-sgRNA control cells (Figure S2C). Thus, the final reporter used for all subsequent experiments contains the 48 bp linker.

To test the feasibility of the reporter in enriching genome-edited cells, Cas9 and reporter co-expressing NALM-6 cells were transduced without or with either *GLuc* control or *LYN*-targeting sgRNA lentiviral vectors (Fig. 1B); to mimic the condition of viral transduction of primary samples, and to aim for a single integration of the lentivirus in the genome^17^, cells were transduced with a low transduction efficiency. Three days after infection, cells were sorted by flow cytometry for either mTagBFP-iRFP double positive or, indicative for Cas9-induced frame shift mutations in the reporter gene, mTagBFP-iRFP-GFP triple-positive cells (Fig. 1B). While around 20% or 40% of cells expressed the sgRNA in control and *LYN* sgRNA transduced cells, respectively, the reporter was edited in less than 3% of the *LYN* KO cell population, as indicated by GFP expression (Fig. 1B). Minor reporter positivity in cells with both Cas9 and the sgRNA in part reflects incomplete gene editing, but also the fact that the reporter is restricted to recognize Indels with a frame shift of − 1 or + 2, while other Indels remain undetected. Capillary immunoassay revealed a dramatic decrease of LYN protein expression in GFP-positive cells as compared to GFP-negative *LYN*-sgRNA-expressing cells or cells expressing the control *GLuc*-sgRNA (Fig. 1C). Importantly, targeting genes located at distinct positions within the reporter demonstrated similar enrichment, indicating that editing efficiency is independent of the position of the targeting sequence within the reporter (Figure S3).

As a next step, we performed droplet digital PCR (ddPCR) to evaluate the percentage of genome-edited alleles at the *LYN* locus in each sub-population (Fig. 1D, Figure S4, Supplemental Table S2)^18^. ddPCR detected a significant enrichment of edited alleles in the reporter-positive subpopulation to around 80% as compared to the reporter-negative population, which contained below 10% edited alleles (Fig. 1D), indicating that cells with gene editing in the reporter revealed reliable gene editing in the genome. These data demonstrate that the reporter system reliably identifies cells with successfully edited loci, enabling their enrichment.

To gain insight into the editing efficiency on a single cell level, Cas9 and reporter co-expressing NALM-6 cells were electroporated with a *LYN*-gRNA (see Methods for details) and single cell cultures were generated from GFP-positive and GFP-negative cells. Electroporation was used to prevent ongoing Cas9 activity, and putatively associated off target effects^19,20^, typical for lentivirally transduced cells with genomic transgene integration. Protein expression analysis demonstrated that 48% (10/21) of GFP-negative clones showed decreased LYN protein expression, while reduced expression of LYN was detected in 95% (21/22) of GFP-positive clones (Fig. 1E and Figure S5A); in 5/22 LYN protein levels were markedly decreased but still readily detectable, which may point to a heterozygous knockout in these clones. Most reporter-positive clones (16/22) demonstrated complete loss of the protein, indicating homozygous gene deletion (Fig. 1E and Figure S5A). Thus, the reporter increased efficiency and reduced the resources required for generating single cell clones, even in straightforward cell lines systems.

To evaluate a functional role for LYN in B-ALL, we expanded 3 of the single cell clones (Figure S5A, bold coloured labels) with putative homozygous (hom) or heterozygous (het) knockout or wildtype (wt) *LYN* genotype and confirmed genotypes by ddPCR (Figure S5B) and protein expression analysis (Fig. 1F, inner panel). In competitive growth assays, no difference in growth behavior between the 3 genotypes was detected after 2 weeks (Figure S6A), indicating that LYN is dispensable for proliferation and survival of NALM-6 cells in vitro. In a next step, we tested response of the single cell clones to conventional chemotherapeutic drugs and detected enhanced response of *LYN*-edited cells to VCR (Fig. 1F), as well as methotrexate and etoposide treatment (Figure S6BC) as compared to *GLuc* controls (ctrl). Interestingly, IC50 of VCR was equally reduced in *LYN* homozygous and heterozygous knockout cells, as compared to wildtype or *GLuc* control cells (Fig. 1F). Similarly, a 50% reduction of LYN protein expression in the heterozygous cells equally sensitized cells to the additional chemotherapeutic drugs methotrexate and etoposide as compared to *LYN*-knockout cells (Figure S6BC). Importantly, the increased drug-sensitivity in the single cell clones was phenocopied when the bulk population of reporter-positive cells was analyzed as compared to *GLuc* controls, reflecting the high enrichment rate of *LYN*-knockout cells from the bulk by the reporter (Figure S7). Thus, *LYN* is an important determinant of response to chemotherapy in NALM-6 cells and even moderate reduction of LYN sensitizes NALM-6 cells to chemotherapy.

**Figure 2:**
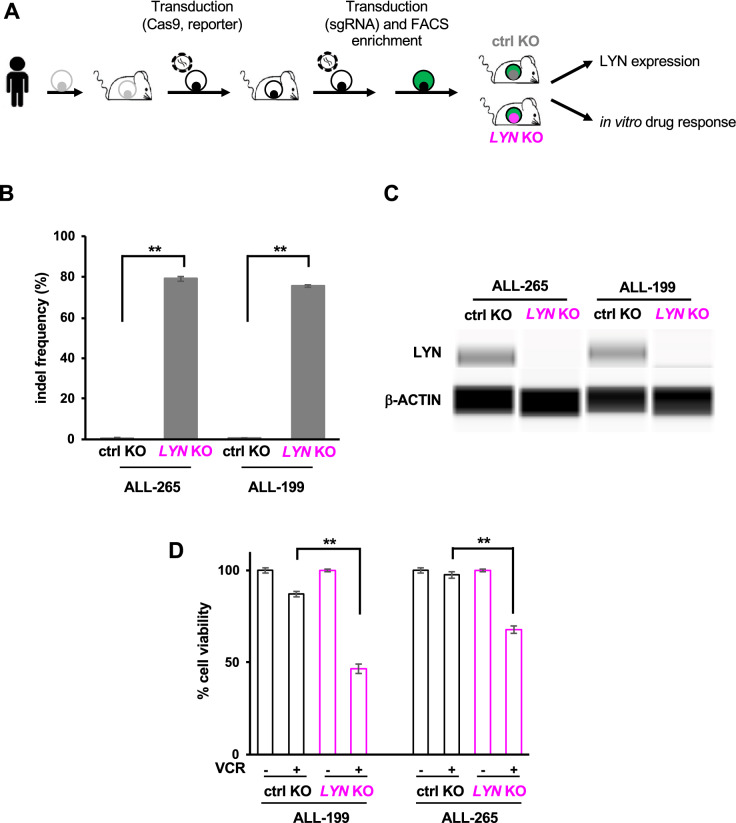
KO of *LYN* sensitizes patient-derived xenograft (PDX) cells towards treatment with Vincristine. (**A**) *Experimental procedure;* Primary pediatric B-ALL cells were amplified in NSG mice, resulting PDX cells were isolated and lentivirally transduced with Cas9 and the reporter expression vector, each followed by one round of amplification in mice. Marker-positive cells, mTaqBFP for Cas9, iRFP for the reporter construct (see Figure S2B for constructs) were transduced with either *GLuc* control or *LYN* sgRNA; reporter GFP-positive PDX cells were injected into NSG mice to generate *GLuc* ctrl (black) and *LYN* KO (pink) PDX derivatives. Mice were sacrificed upon advanced leukemia and KO cells subjected to LYN protein expression analysis and in vitro drug treatment. (**B**) *Enrichment of indel frequency at the LYN locus in PDX cells;* Digital droplet PCR (ddPCR) was performed to determine percentage of *LYN* edited alleles in cells with control KO versus *LYN* KO from PDX ALL-265 and ALL-199. Mean ± SD of 3 independent experiments is shown; **p < 0.01. (**C**) *LYN protein expression is reduced in reporter positive PDX cells;* Automated calculated pictures of capillary immunoassay of LYN protein expression in cells sorted as described in (**A**). β-ACTIN served as a loading control; one representative calculated picture out of 3 independent experiments is shown. (**D**) *LYN knockout sensitizes PDX cells to vincristine (VCR) treatment;* PDX ALL-265 and ALL-199 *GLuc* KO (ctrl) or *LYN* KO cells were treated with or without 1 ng/ml VCR in vitro for 2 days. Cell viability was determined by flow cytometry. Mean ± SD of 3 independent experiments is shown. **p < 0.01.

Having established the surrogate reporter, we next tested our system in two PDX ALL models, ALL-199 and ALL-256 (patient characteristics are detailed in Supplemental Table S3 as published previously^21–23^). Compared to leukemia cell lines, PDX ALL cells are substantially more challenging in handling as they are hard to transduce and reluctant to grow in vitro^21,22^. We sequentially transduced PDX cells first with a Cas9 expression plasmid and second with the reporter, each followed by one round of enrichment and amplification of transduced cells in mice (constructs are detailed in Figs. 1A and S2B). We have previously analyzed clonal stability of PDX models after lentiviral transduction and amplification in mice, and did not find evidence of major clonal selection^24^, suggesting that the sequential enrichment steps may not drastically affect the overall mutational pattern of PDX samples. In a third round, PDX cells were transduced with the *LYN* sgRNA or control *GLuc* sgRNA vectors. 5 days after sgRNA transduction, PDX cells were enriched for the scarce population of reporter positive cells, which were re-transplanted into next recipient mice (Figs. 2A, S2B, S8). GFP-positive *LYN* and *GLuc* sgRNA expressing PDX cells displayed a similar expansion rate in mice (Supplemental Table S4), indicating that LYN does not significantly affect growth of PDX cells in vivo in accordance with the in vitro observation in NALM-6 cells.

PDX cells were re-isolated after passaging and frequency of genome-edited alleles and LYN protein expression was analyzed in GFP-positive cells of *LYN* KO compared to *GLuc* control PDX cells. These experiments revealed that the *LYN* locus was edited in around 80% of cells (Fig. 2B), which is associated with a substantial decrease of LYN protein expression (Fig. 2C), demonstrating efficient enrichment of edited PDX cells by the use of the surrogate reporter. In a next step, therapy response of PDX cells was evaluated in short term in vitro cultures. Compared to control *GLuc* KO PDX cells, *LYN* KO PDX cells were significantly more sensitive to VCR treatment in both ALL PDX models tested (Fig. 2D), reproducing the phenotype of NALM-6 cells in two PDX ALL models.

Our study provided a clinically relevant genetic model to study LYN, a SRC-family kinase that is often activated in B-ALL by rare genetic and non-genetic mechanisms and has been associated with treatment response^8–15,25^. Our data indicate that LYN regulates therapy response of preB-ALL cell lines and PDX cells. Further studies are needed to evaluate whether available drugs that target LYN^26,27^ can improve response to conventional therapy in relapsed B-ALL.

Taken together, our reporter system strongly enriches cells with successful Cas9-induced gene-editing from the large pool of non-edited cells. By using the surrogate reporter, we successfully selected KO clones and extended the use of CRISPR/Cas9 gene editing to PDX cells. Despite the fact that the reporter system may overestimate editing efficiency, e.g. due to differences in chromatin state at the genomic loci versus the targeting site in the reporter^28^, we demonstrate here that our reporter was highly specific to enrich for true KO cells. Application of the reporter system will enable extending the use of CRISPR–Cas9-mediated gene editing to cells which are difficult to handle, but represent highly clinically relevant and precious preclinical model systems.

## Materials and methods

### Plasmid cloning

The reporter plasmid is based on a pCDH lentiviral vector which expressed iRFP720 followed by T2A under control of the Elongation factor 1-alpha (EF1α) promoter. The fragment contains several Cas9:sgRNA target sequences (see supplementary Table S1, right panel) followed by a GC-rich sequence (GGGGSLVPRGSGGGGS) and was purchased from IDT Inc. (Coralville, IA, USA); in its 3′ region, the fragment contains the sequence of destabilized GFP^29^ with a frame shift of + 1 base. The fragment was cloned into the pCDH vector using NotI and SalI. Details on the sequences are described in Figure S1.

Cloning of the the pCDH-based lentiviral sgRNA expression vector, with mTagBFP or mCherry expressed under the control of the EF1α promoter was previously described^23^. The sgRNA sequences (see supplementary Table S1, left panel) were synthesized (Eurofins MWG-Operon, Ebersberg, Germany), annealed and ligated into the vector using BbsI.

For Cas9 expression, a DNA fragment containing the cDNA of spleen focus forming virus (SFFV) promoter (Addgene plasmid #27348), humanized Staphyococcus pyogenes Cas9 (hSpCas9 PX330, Addgene plasmid #42230) and a T2A-linked mTaqBFP fluorochrome (pTagBFP-C vector, PF171, Envrogen, Lawrenceville, NJ) or a truncated form of the human nerve growth factor receptor (hNGFR) was amplified by PCR and cloned into the pCDH vector (Addgene Plasmid #72263) as previously described^23^.

### Ethical statements

Prior to obtaining the two primary B-ALL patient samples, written informed consent was obtained from all patients or from parents/caregivers in cases in which patients were minors. The study was performed in accordance with the ethical standards of the responsible committee for human experimentation (written approval by the Ethikkommission des Klinikums der Ludwig-Maximilians-Universität Munich, number 068-08 and 222-10) and with the Helsinki Declaration of 1975, as revised in 2000.

Animal trials were performed in compliance with the ARRIVE guidelines (https://arriveguidelines.org) and in accordance with the current ethical standards of the official committee on animal experimentation (written approval by the Regierung von Oberbayern, tierversuche@reg-ob.bayern.de; July 2010, number 55.2-1-54-2531-95-10; July 2010, 55.2-1-54-2531.6-10-10; January 2016, ROB-55.2 Vet-2532. Vet_02-15-193; May 2016, ROB-55.2 Vet-2532. Vet_02-16-7 and August 2016, ROB-55.2 Vet-2532.Vet_03-16-56).

### In vitro culture conditions and drug treatment

The human B cell precursor leukemia cell line NALM-6 (ACC128) was purchased from DSMZ (German collection of microorganisms and cells, Braunschweig, Germany) and maintained in RPMI-1640 supplemented with 2 mM l-glutamine (Life Technologies GmbH, Darmstadt, Germany) and 10% FCS (Biochrom AG, Berlin, Germany). HEK293T cells were maintained in DMEM (Life Technologies) supplemented with 2 mM l-glutamine and 10% FCS. PDX cells were cultured in RPMI medium supplemented with 20% FSC, 1% pen/strep, 1% gentamycin, 6 mg/l insulin, 3 mg/l transferrin, 4 μg/l selenium (Gibco, San Diego, USA), 2 mM glutamine, 1 mM sodium pyruvate, 50 μM α-thioglycerol (Sigma-Aldrich, St. Louis, USA). For drug treatments, Vincristine (VCR, Merck, Darmstadt, Germany), Etoposide (Merck) or Methotrexate (Merck) was added to the culture medium. Cell viability was detected by LSRII (BD Bioscience, San Jose, CA) based on FCS/SSC gating at the end of the experiment.

### Lentiviral transduction and enrichment of NALM-6 and PDX ALL cells

PDX ALL-265 and ALL-199 were established and the NOD.Cg-Prkdcscid Il2rgtm1Wjl/SzJ (NSG) mouse model performed as described^21–23^; Lentiviruses were produced and cells infected as described^21–23^; in PDX and NALM-6 cells, when creating the stable *LYN* KO cell lines, transduction efficiency was at or below 40% to aim for single integrations of lentiviruses into the host genome^17^. After lentiviral transduction, PDX cells were injected intravenously into NSG mice for amplification 24 h post infection, or maintained in vitro 4 days and enriched for transgene expression before injection. NALM-6 or re-isolated PDX cells from the 2 donor mice (*Gluc* and *LYN*) were analyzed and sorted by flow cytometry for fluorochrome expression (LSRII and FACSAria III from Becton Dickinson San Jose, CA, USA) or enriched using magnetic cell separation (MACS) targeting NGFR (Miltenyi Biotech, Bergisch Gladbach, Germany) as previously described^21,22^.

### Transient gRNA transfection and single cell colony

For electroporation, the gRNA complex was prepared according to the manufacturer’s recommendations by mixing Alt-R CRISPR–Cas9 crRNA (1 µM) and Alt-R transactivating crRNA (tracrRNA) coupled to ATTO 550 (IDT Inc.) to a final duplex concentration of 44 µM. The mixture was heated to 95 °C for 5 min and cooled down to room temperature for at least 60 min. For electroporation, 1.2 million Cas9 and reporter co-expressing cells were re-suspended in 120 µl R buffer and 4.9 µl of 44 µM gRNA mixture were added. 100 µl were subjected to each electroporation using the Neon device (Invitrogen, Eugene, OR, USA) under optimized conditions (1350 V, 10 ms, 3 pulses).

5 days after transient transfection of the *LYN* gRNA, single cells were sorted each in one well of a 96-well plate by FACSAria III according to GFP expression. Cells were allowed to expand for 2–3 weeks before they were transferred to 12-well plates and used for further analysis.

### Capillary protein immunoassay for protein expression analysis

Flow cytometry-enriched cell populations were incubated in lysis buffer (#9803, Cell Signaling Technology, Boston, USA) and PMSF (1:200) on ice for 30 min. Protein concentration was measured by BCA assay (#7780, New England Biolabs, Beverly, USA) and abundance of specific proteins determined by capillary immunoassay. Procedures were performed following manufacturer’s instructions; in brief, each capillary was loaded with protein lysate and electrophoresis was performed; capillaries were processed to attach all proteins to the capillary wall and incubated with a single antibody; results were measured as emission curves from each capillary and “Western-Blot-like presentations” calculated thereof using the Compass software (ProteinSimple), including quantification. Final “Western-Blot-like presentations” appear clearly different from conventional Western Blots as described^23^; due to very high sensitivity, equal loading is hard to achieve, but also not required, as protein amounts are calculated with high sensitivity and reliability. Primary antibody against LYN was purchased from R&D systems (AF3206, Minneapolis, MN, USA), FLAG from R&D systems (MAB8529, Minneapolis, MN, USA) and β-ACTIN from Novus biologicals (NB600-501SS, Littleton, CO, USA).

### Digital droplet PCR (ddPCR)

The ddPCR assay was performed as previously described^18,30^; in brief, the following reaction mix was prepared (final concentrations in 20 µl total reaction volume): ddPCR SuperMix for Probes (no dUTP) (1 ×, Bio-Rad, Richmond, CA, USA), forward primer (900 nM), reverse primer (900 nM), reference probe (FAM 250 nM), NHEJ/drop-off probe (HEX, 250 nM), nuclease-free water, and 50–100 ng genomic DNA. All primers and probes were designed using Primer3 plus (http://primer3plus.com) and purchased from Eurofins MWG-Operon (Ebersberg, Germany). All probes included the BHQ1 quencher. All ddPCR assays were analyzed using the QX200 droplet reader and Quantasoft software version 1.7.4 (Bio-Rad). Standard ddPCR thermal cycling conditions were with an annealing temperature of 55 °C. All primers are described in supplementary Table S3.

### Statistical analysis

Two-tailed Student’s t test was used to compare the difference of groups. *P* value less than 0.05 was considered statistically significant.

## Supplementary Information

Supplementary Information.

## References

[1] Ren C, Xu K, Segal DJ, Zhang Z (2019). Strategies for the enrichment and selection of genetically modified cells. Trends Biotechnol..

[2] Milan T, Canaj H, Villeneuve C, Ghosh A, Barabé F, Cellot S (2019). Pediatric leukemia: moving toward more accurate models. Exp. Hematol..

[3] Pabst C, Krosl J, Fares I, Boucher G, Ruel R, Marinier A (2014). Identification of small molecules that support human leukemia stem cell activity ex vivo. Nat. Methods.

[4] Stafman LL, Williams AP, Marayati R, Aye JM, Markert HR, Garner EF (2019). Focal adhesion kinase inhibition contributes to tumor cell survival and motility in neuroblastoma patient-derived xenografts. Sci. Rep..

[5] Terwilliger T, Abdul-Hay M (2017). Acute lymphoblastic leukemia: a comprehensive review and 2017 update. Blood Cancer J..

[6] Kim H, Um E, Cho SR, Jung C, Kim H, Kim JS (2011). Surrogate reporters for enrichment of cells with nuclease-induced mutations. Nat. Methods.

[7] Ramakrishna S, Cho SW, Kim S, Song M, Gopalappa R, Kim JS (2014). Surrogate reporter-based enrichment of cells containing RNA-guided Cas9 nuclease-induced mutations. Nat. Commun..

[8] Gang EJ, Kim HN, Hsieh Y-T, Ruan Y, Ogana HA, Lee S (2020). Integrin α6 mediates the drug resistance of acute lymphoblastic B-cell leukemia. Blood.

[9] Dai HP, Yin J, Li Z, Yang CX, Cao T, Chen P (2020). Rapid molecular response to dasatinib in a pediatric relapsed acute lymphoblastic leukemia with NCOR1-LYN fusion. Front. Oncol..

[10] Imamura T, Kiyokawa N, Kato M, Imai C, Okamoto Y, Yano M (2016). Characterization of pediatric Philadelphia-negative B-cell precursor acute lymphoblastic leukemia with kinase fusions in Japan. Blood Cancer J..

[11] Reshmi SC, Harvey RC, Roberts KG, Stonerock E, Smith A, Jenkins H (2017). Targetable kinase gene fusions in high-risk B-ALL: a study from the Children's Oncology Group. Blood.

[12] Yano M, Imamura T, Asai D, Kiyokawa N, Nakabayashi K, Matsumoto K (2015). Identification of novel kinase fusion transcripts in paediatric B cell precursor acute lymphoblastic leukaemia with IKZF1 deletion. Br. J. Haematol..

[13] Erasmus MF, Matlawska-Wasowska K, Kinjyo I, Mahajan A, Winter SS, Xu L (2016). Dynamic pre-BCR homodimers fine-tune autonomous survival signals in B cell precursor acute lymphoblastic leukemia. Sci. Signal..

[14] Geng H, Hurtz C, Lenz KB, Chen Z, Baumjohann D, Thompson S (2015). Self-enforcing feedback activation between BCL6 and pre-B cell receptor signaling defines a distinct subtype of acute lymphoblastic leukemia. Cancer Cell.

[15] Heckl D, Kowalczyk MS, Yudovich D, Belizaire R, Puram RV, McConkey ME (2014). Generation of mouse models of myeloid malignancy with combinatorial genetic lesions using CRISPR–Cas9 genome editing. Nat. Biotechnol..

[16] Roidos P, Sungalee S, Benfatto S, Serçin Ö, Stütz AM, Abdollahi A (2020). A scalable CRISPR/Cas9-based fluorescent reporter assay to study DNA double-strand break repair choice. Nat. Commun..

[17] Charrier S, Ferrand M, Zerbato M, Précigout G, Viornery A, Bucher-Laurent S (2011). Quantification of lentiviral vector copy numbers in individual hematopoietic colony-forming cells shows vector dose-dependent effects on the frequency and level of transduction. Gene Ther..

[18] Findlay SD, Vincent KM, Berman JR, Postovit LM (2016). A digital PCR-based method for efficient and highly specific screening of genome edited cells. PLoS ONE.

[19] Rayner E, Durin M-A, Thomas R, Moralli D, O'Cathail SM, Tomlinson I (2019). CRISPR–Cas9 causes chromosomal instability and rearrangements in cancer cell lines, detectable by cytogenetic methods. CRISPR J..

[20] Rezza A, Jacquet C, Le Pillouer A, Lafarguette F, Ruptier C, Billandon M (2019). Unexpected genomic rearrangements at targeted loci associated with CRISPR/Cas9-mediated knock-in. Sci. Rep..

[21] Terziyska N, Castro Alves C, Groiss V, Schneider K, Farkasova K, Ogris M (2012). In vivo imaging enables high resolution preclinical trials on patients' leukemia cells growing in mice. PLoS ONE.

[22] Ebinger S, Ozdemir EZ, Ziegenhain C, Tiedt S, Castro Alves C, Grunert M (2016). Characterization of rare, dormant, and therapy-resistant cells in acute lymphoblastic leukemia. Cancer Cell.

[23] Liu W-H, Mrozek-Gorska P, Wirth A-K, Herold T, Schwarzkopf L, Pich D (2020). Inducible transgene expression in PDX models in vivo identifies KLF4 as a therapeutic target for B-ALL. Biomark. Res..

[24] Vick B, Rothenberg M, Sandhofer N, Carlet M, Finkenzeller C, Krupka C (2015). An advanced preclinical mouse model for acute myeloid leukemia using patients' cells of various genetic subgroups and in vivo bioluminescence imaging. PLoS ONE.

[25] Hu Y, Liu Y, Pelletier S, Buchdunger E, Warmuth M, Fabbro D (2004). Requirement of Src kinases Lyn, Hck and Fgr for BCR-ABL1-induced B-lymphoblastic leukemia but not chronic myeloid leukemia. Nat. Genet..

[26] Tasian SK, Loh ML, Hunger SP (2017). Philadelphia chromosome-like acute lymphoblastic leukemia. Blood.

[27] Leonard JT, Rowley JSJ, Eide CA, Traer E, Hayes-Lattin B, Loriaux M (2016). Targeting BCL-2 and ABL/LYN in Philadelphia chromosome–positive acute lymphoblastic leukemia. Sci. Transl. Med..

[28] Verkuijl SAN, Rots MG (2019). The influence of eukaryotic chromatin state on CRISPR–Cas9 editing efficiencies. Curr. Opin. Biotechnol..

[29] Li X, Zhao X, Fang Y, Jiang X, Duong T, Fan C (1998). Generation of destabilized green fluorescent protein as a transcription reporter. J. Biol. Chem..

[30] Rose JC, Stephany JJ, Valente WJ, Trevillian BM, Dang HV, Bielas JH (2017). Rapidly inducible Cas9 and DSB-ddPCR to probe editing kinetics. Nat. Methods.

