# Supplementary material for: A reporter system for enriching CRISPR/Cas9 knockout cells in technically challenging settings like patient models
Source: Sci Rep. 2021 Jun 16;11:12649. doi: 10.1038/s41598-021-91760-9 (PMC8209181; doi:10.1038/s41598-021-91760-9)
Supplement: Supplementary file 1 — Supplementary Information. [file 41598_2021_91760_MOESM1_ESM.pdf]

Supplemental information

A reporter system for enriching CRISPR/Cas9 knockout cells in technically challenging settings like patient models

Wen-Hsin Liu, Kerstin Völse, Daniela Senft and Irmela Jeremias

This file contains:

Supplemental Tables

Supplemental Figures and Legends

Supplemental Tables

Table S1 sgRNA sequence and targeting sequence

Position	Target gene	sgRNA sequence (incl PAM)
1	***FERMT3***	AGGCGCAGTGCGCGGCGGTTGGG
2	***GLuc***	GGACTCTTTGTCGCCTTCGTAGG
3	*PLK1*	TCCGGGATCTCTTTCGCCGGTGG
4	***XIAP***	CCTTCACCTACGAAAACACACAG
5	*BTRC*	CCTTGATTGTGTTGTCTCGAAGG
6	*MCL1*	GGAGACCTTACGACGGGTTGGG
7	*RPA3*	CCCAGGTCGCGCATCAACGCCGG
8	*HIF1A*	TTCTTTACTTCGCCGAGATCTGG
9	*TNFRSF1A*	CCTCAATGGGACCGTGCACCTGT
10	*TP53*	CCATGAGCGCTGCTCAGATAGCG
11	*ADAM10*	TCTGCTCCTCTCCTGGGCGGCGG
12	***ADAM17***	CCTCCGGATGACCCGGGCTTCG
13	*CD44*	CCGTCCGAGAGATGCTGTAGCGA
14	*CXCR4*	CCACCAGAAGCGCAAGGCCCTCA
15	*RUNX1*	CCAGCGGCAACGCCTCGCTCATC
16	***LYN***	TGAAAGACAAGTCGTCCGGGTGG
17	*TNFRSF1B*	CCCAGACGGCGACGGGCGCCATG

Bold font indicates genes of interest that were targeted within this study.

Table S2 ddPCR primer sequence

lyn mismatch fw	5’ CTTCTAACATGCGGTGACGTG 3’
lyn mismatch rv	5’ CTTGCTCACCATGGATCCTTC 3’
lyn ddPCR fw	5’ GATCTTAGCTTCGCCATCCT 3’
lyn ddPCR rv	5’ ATGGCGGACTTGAAGAAGTC 3’
lyn ddPCR reference probe	5’ CACATGTCCTTCTTCCAGTTG 3’
lyn ddPCR target probe	5’ CTTTGGCTCTATTCCTTCCAC 3’

Table S3. Clinical characteristics of ALL patients

sample	disease stage*	age [years]	sex	cytog-enetics	mutations and gene fusions∞
**ALL-199**	R2	8	f	somatic trisomy21; leukemic homozygous 9p deletion	P2RY8-CRLF2
**ALL-265**	R1	5	f	hyperdipoidy with additional 6,13,14,17,18,21,X chromosome	KMT2D, HERC1

*when the primary sample was obtained; ∞ mutations determined by panel sequencing for 100 genes recurrently mutated in ALL; R1 = 1st relapse; R2 = 2nd relapse; f = female; N.D. not determined.

Table S4 PDX cells expansion time

	ALL-199		ALL-265	
sgRNA	*GLuc*	*LYN*	*GLuc*	*LYN*
Number of cell injected	160,000	115,000	100,000	100,000
Days of tumor growth	42	46	50	54

Supplemental Figures

Figure S1

A cDNA sequence

iRFP::: GAA GAG GCG GCC GCT GGC GGA GGC GGT TCT CTT GTA CCA AGA GGC TCA GGC
GGA GGC GGT ATG CAT AAT GGG AAG GCG CAG TGC GCG GCG GTT GGG AGG ACT CTT TGT
CGC CTT CGT AGG TCC GGG ATC TCT TTC GCC GGT GGA CCT TCA CCT ACG AAA ACA CAC
AGC CTT GAT TGT GTT GTC TCG AAG GTG GAG ACC TTA CGA CGG GTT GGG CCC AGG TCG
CGC ATC AAC GCC GGT TCT TTA CTT CGC CGA GAT CTG GCC TCA ATG GGA CCG TGC ACC
TCT AAC CAT GAG CGC TGC TCA GAT AGC GTC TGC TCC TCT CCT GGG CGG CGG CCT CCG
GAT GAC CCG GGC TTC GGA CCG TCC GAG AGA TGC TGT AGC GAC CAC CAG AAG CGC AAG
GCC CTC ACC AGC GGC AAC GCC TCG CTC ATC AAT GAA AGA CAA GTC GTC CGG GTG GCC
CAG ACG GCG ACG GGC GCC ATG CAC CGG TGG CGG AGG CGG TTC TCT TGT ACC AAG AGG
CTC AGG CGG AGG CGG TTC TGC GGA AGC GGG AGG ATC CAT GGT GAG CAA GGG CGA GGA
GCT GTT CAC CGG GGT GGT GCC CAT CCT GGT CGA GCT GGA CGG CGA CGT AAA CGG CCA
CAA GTT CAG CGT GTC CGG CGA GGG CGA GGG CGA TGC CAC CTA CGG CAA GCT GAC CCT
GAA GTT CAT CTG CAC CAC CGG CAA GCT GCC CGT GCC CTG GCC CAC CCT CGT GAC CAC
CCT GAC CTA CGG CGT GCA GTG CTT CAG CCG CTA CCC CGA CCA CAT GAA GCA GCA CGA
CTT CTT CAA GTC CGC CAT GCC CGA AGG CTA CGT CCA GGA GCG CAC CAT CTT CTT CAA
GGA CGA CGG CAA CTA CAA GAC CCG CGC CGA GGT GAA GTT CGA GGG CGA CAC CCT GGT
GAA CCG CAT CGA GCT GAA GGG CAT CGA CTT CAA GGA GGA CGG CAA CAT CCT GGG GCA
CAA GCT GGA GTA CAA CTA CAA CAG CCA CAA CGT CTA TAT CAT GGC CGA CAA GCA GAA
GAA CGG CAT CAA GGT GAA CTT CAA GAT CCG CCA CAA CAT CGA GGA CGG CAG CGT GCA
GCT CGC CGA CCA CTA CCA GCA GAA CAC CCC CAT CGG CGA CGG CCC CGT GCT GCT GCC
CGA CAA CCA CTA CCT GAG CAC CCA GTC CGC CCT GAG CAA AGA CCC CAA CGA GAA GCG
CGA TCA CAT GGT CCT GCT GGA GTT CGT GAC CGC CGC CGG GAT CAC TCT CGG CAT GGA
CGA GCT GTA CAA GAA GCT TAG CCA TGG CTT CCC GCC GGA GGT GGA GGA GCA GGA TGA
TGG CAC GCT GCC CAT GTC TTG TGC CCA GGA GAG CGG GAT GGA CCG TCA CCC TGC AGC
CTG TGC TTC TGC TAG GAT CAA TGT GTA GGT CGA C

Restriction enzyme cutting site; T2A; **A/AA/C** linker to avoid stop codon; Glycine linker; des GFP
▼ site of putative frameshift

B Prediction of protein translation

Reporter out of frame (without editing)

MHNGKAQCAAVGRTLCRLRRSGISFAGGPSPTKTHSLDCVVSKVETLRRVGPRSRINAGSLLRRDLASMGPCTSN
HERCSDSVCSSPGRRPPDDPGFGPSERCCSDHQKRKALTSGNASLINERQVVRVAQTATGAMHRWRRRFSCTKRL
RRRRFCGSGRIHGEQGRGAVHRGGAHPGRAGRRRKRPQVQRVRRGRGRCHLRQADPEVHLHHRQAARALAHPRDH
PDLRRAVLQPLPRPHEAARLLQVRHARRLRPGAHHLLQGRRQLQDPRRGEVRGRHPGEPHRAEGHRLQGGRQHPG
AQAGVQLQQPQRLYHGRQAEERHQGELQDPPQHRGRQRAARRPLPAEHPHRRRPRAAARQPLPEHPVRPEQRPQR
EARSHGPAGVRDRRRDHSRHGRAVQEA*PWLPAGGGGAG*WHAAHVLCPGERDGPSPCSLCFC*DQCVGR

Reporter after frameshift mutation (+1/-2)

▼
MHKWEGAVRGGWEDSLSPS*VRDLFRRWTFTYENTQP*LCCLEGGDLTTGWAQVAHQRRFFTSPRSGLNGTVHL*
P*ALLR*RLLLSWAAASG*PGLRTVREML*RPPEAQGPHQRQRLAHQ*KTSRPGGPDGDGRHAPVAEAVLLYQEA
QAEAVLRKREDPW*ARARSCSPGWCPSWSSWTAT*TATSSACPARARAMPPTAS*P*SSSAPPASCPCPGPPS*P
P*PTACSASAATPTT*SSTTSSSPPCPKATSRSAPSSSRTTATTRPAPR*SSRATPW*TASS*RASTSRRTATSW
GTSWSTTTTATTSISWPTSRRTASR*TSRSATTSRTAACSSPTTTSRTPPSATAPCCCPTTTT*APSPP*AKTPT
RSAITWSCWSS*PPPGSLSAWTSCTRSLAMASRRRWRSRMMARCPCLVPRRAGWTVTLQPVLLLGSMCRS

Reporter after frameshift mutation (+2/-1)

▼
MHKMGRRSARRLGGLFVAFVGPGSLSPVDLHLRKHTALIVLSRRWRPYDGLGPGRASTPVLYFAEIWPQWDRAPL
TMSAAQIASAPLLGGGLRMTRASDRPRDAVATTRSARPSPAATPRSSMKDKSSGWPRRRRAPCTGGGGGSLVPRG
SGGGGSAEAGGSMVSKGEELFTGVVPILVELDGDVNGHKFSVSGEGEGDATYGKLTLKFICTTGKLPVPWPTLVT
TLTYGVQCFSRYPDHMKQHDFFKSAMPEGYVQERTIFFKDDGNYKTRAEVKFEGDTLVNRIELKGIDFKEDGNIL
GHKLEYNYNSHNVYIMADKQKNGIKVNFKIRHNIEDGSVQLADHYQQNTPIGDGPVLLPDNHYLSTQSALSKDPN
EKRDHMVLLEFVTAAGITLGMDELYKKLSHGFPPEVEEQDDGTLPMSCAQESGMDRHPAACASARINV*

Figure S1 Detailed design of the Cas9 reporter system (related to Fig. 1A)

A) cDNA sequence of the reporter. Based on a previously published reporter (Kim et al., 2011), two fluorochrome markers under the control of a single constitutive promoter, connected by a 2A peptide for posttranscriptional processing was used. The upstream fluorochrome, the near-infrared fluorescent protein (iRFP) 720 (purple), was stably expressed indicating presence of the reporter in the target cell. The second fluorochrome, destabilized green fluorochrome protein (desGFP, green), was cloned out-of-frame and is not expressed from the unedited reporter. Between the 2 fluorophores, 17 Cas9:sgRNA target sites, consisting of 20bp of the genomic sequence of the gene of interest (GOI) including its 5′-NGG-3′ protospacer adjacent motif (PAM) and an 48 bp GC-rich linker (brown color) was cloned.

B) Predicted protein expression of the reporter. Predicted protein expression from the reporter without editing (upper panel), and with distinct frameshifts (red arrow) upon editing (middle and lower panel) are depicted. GFP is only expressed, if any of the Cas9:sgRNA target sites in the reporter were cleaved and a -1/4/7/10… insertion or +2/5/8/11… deletion was induced by NHEJ (lower panel). Other frameshift mutations (i.e. +1/-2) will not induce GFP expression. * = stop codon

Figure S2

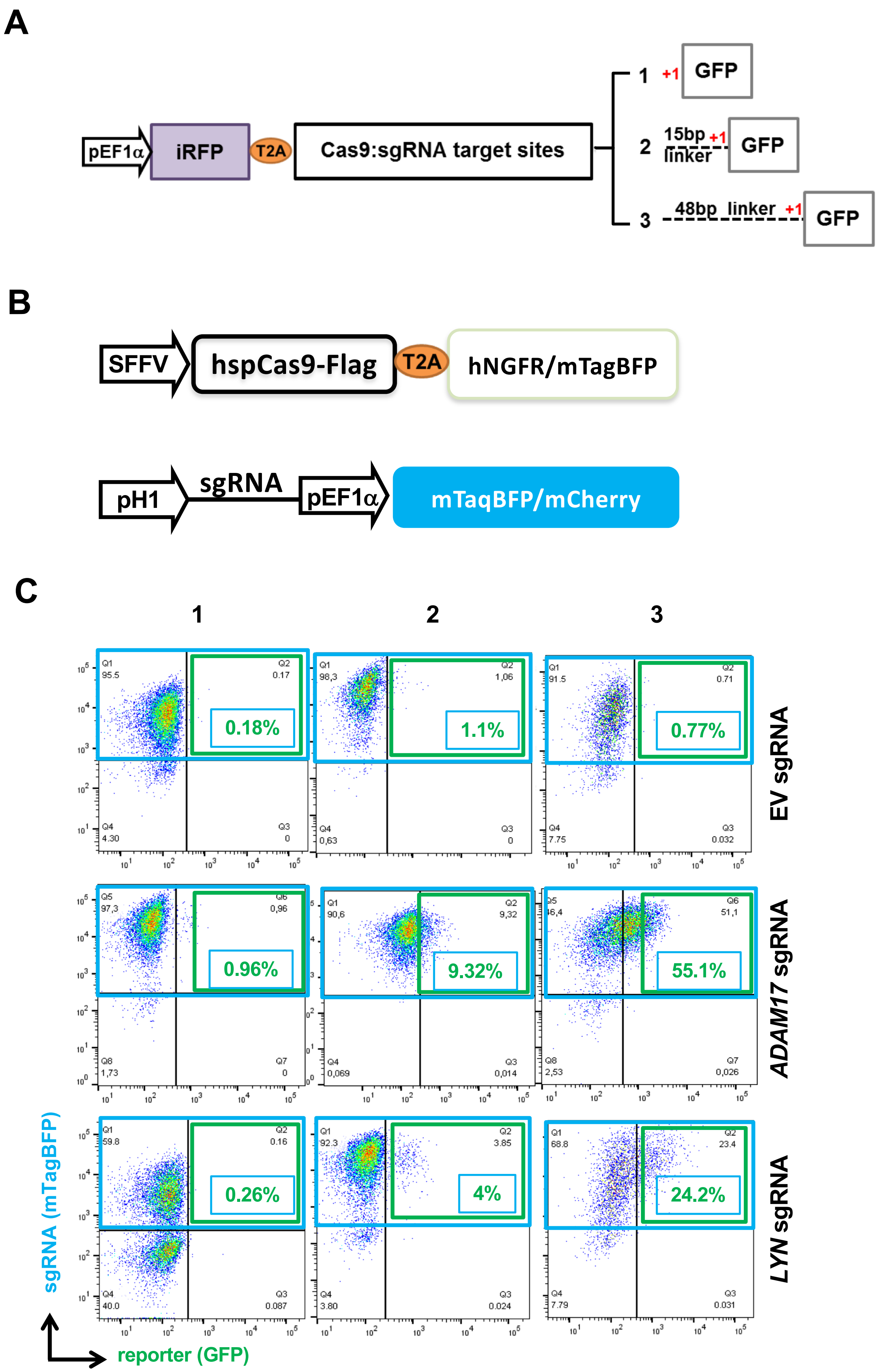
A
pEF1α
iRFP
T2A
Cas9:sgRNA target sites
1
+1
GFP
2
15bp linker
+1
GFP
3
48bp linker
+1
GFP
B
SFFV
hspCas9-Flag
T2A
hNGFR/mTagBFP
pH1
sgRNA
pEF1α
mTaqBFP/mCherry
C
1
2
3
0.18%
1.1%
0.77%
EV sgRNA
0.96%
9.32%
55.1%
ADAM17 sgRNA
0.26%
4%
24.2%
LYN sgRNA
sgRNA (mTagBFP)
reporter (GFP)

Figure S2 The GC-linker enhances marker expression in gene-edited cells

A) Scheme of the reporter with or without a GC-rich linker. The Cas9:sgRNA target sites was linked either 1) directly to the GFP marker, or 2) a 15bp, or 3) a 48bp GC-rich linker was cloned between the Cas9:sgRNA target site and the GFP marker.

B) Scheme of constructs used to generate knockout cells
Upper panel: Flag-tagged human codon-optimized *S. pyogenes* (hsp)Cas9 was constitutively expressed from the spleen focus-forming virus (SFFV) promoter together with a truncated form of the human nerve growth factor receptor (hNGFR) or mTagBFP, linked via a virus–derived 2A auto-cleavage site (T2A).
Lower panel: The single guide (sg)RNAs were expressed under control of the H1 Pol III promoter. Expression of mTagBFP or mCherry from the EF1a promoter allowed enrichment of transgenic cells.

C) A longer linker allows better expression of GFP. Cas9-expressing NALM-6 cells were lentivirally transduced with an empty sgRNA vector (EV-sgRNA) as a control or the *ADAM17*-sgRNA or *LYN*-sgRNA vector together with mTagBFP and the three different reporter constructs described in **A**. After 3 days, cells were harvested and analyzed by flow cytometry. The ratio of sgRNA-edited GFP^{+} cells (green frame) to all sgRNA expressing cells (blue frame) is indicated in percent. One out of three independent experiments is shown.

Figure S3

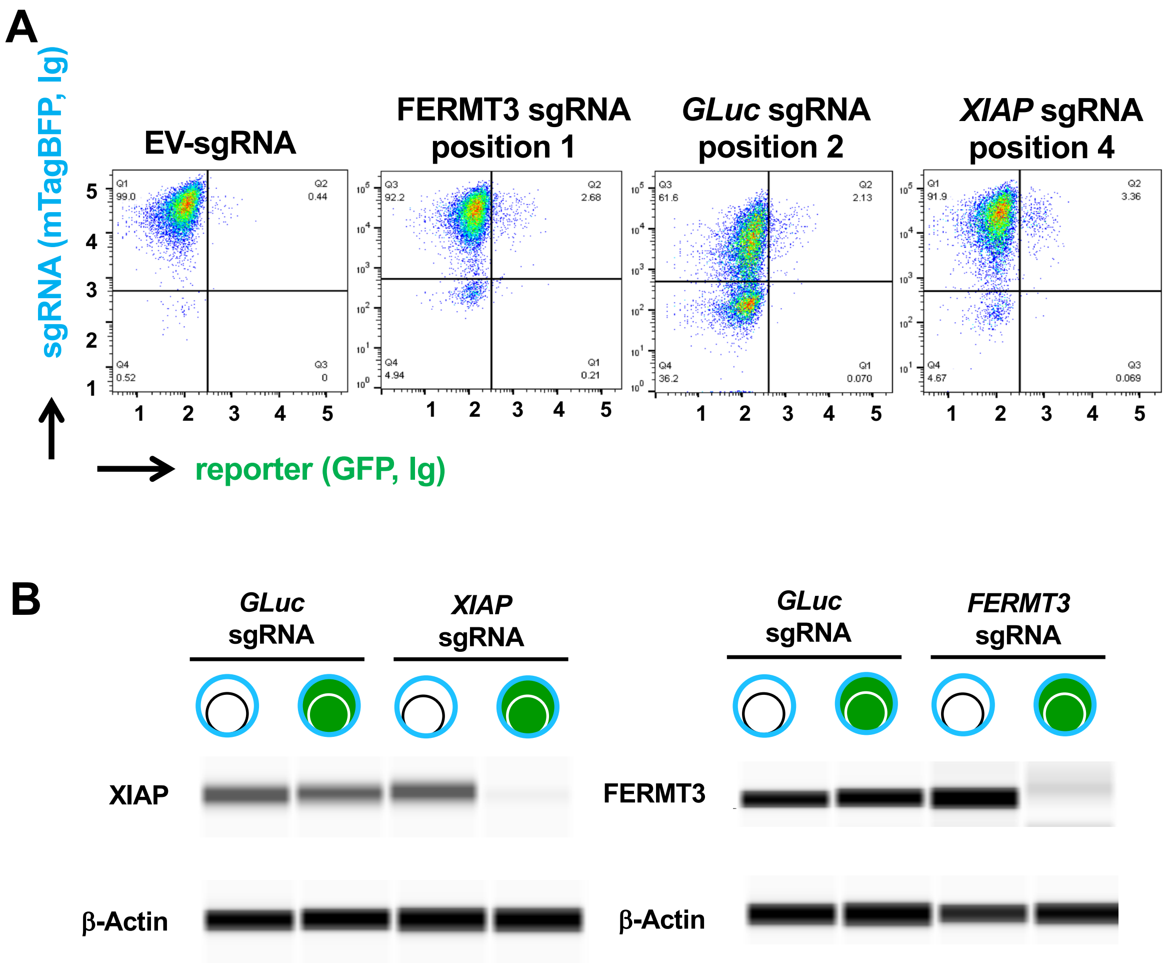

Figure S3 Enrichment of gene-edited cells independent of the location of the Cas9:sgRNA targeting site

A) Cas9-expressing NALM-6 cells were lentivirally transduced with an empty sgRNA vector (EV-sgRNA) as a control or the *FERMT3*-sgRNA, *GLuc*-sgRNA or *XIAP*-sgRNA vector together with the reporter constructs. After 3 days, cells were harvested and analyzed by flow cytometry. One out of three independent experiments is shown.

B) Automated calculated pictures of capillary immunoassay of XIAP and FERMT3 protein expression in cells sorted as in A. b-ACTIN served as loading control; one representative calculated picture out of 3 independent experiments is shown.

Figure S4

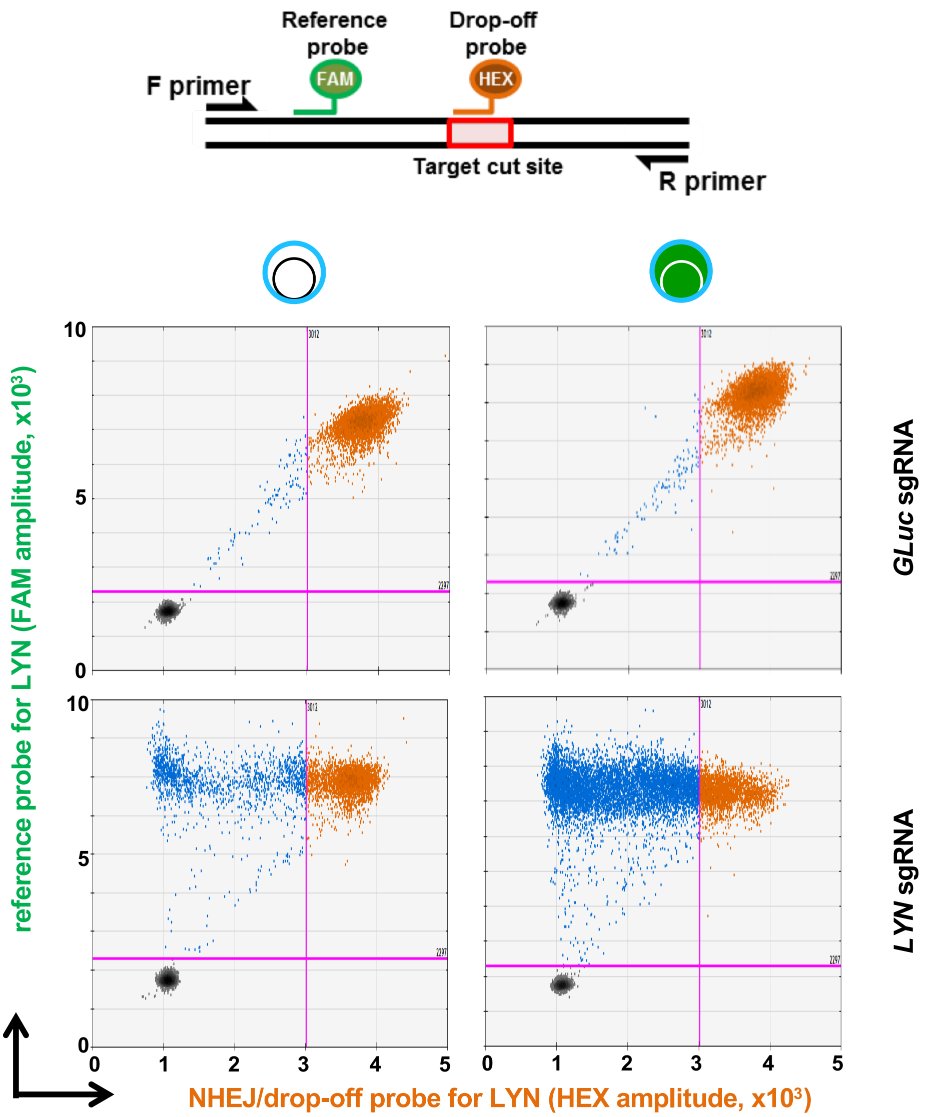

Figure S4 Raw data of the digital droplet (dd)PCR (related to Figure 1D).

Upper panel: scheme of the ddPCR drop-off assay.

Lower panel: Raw data related to Figure **1D**; x-axis indicates drop-off probe coupled to HEX, y-axis indicates reference probe coupled to FAM; upper left quadrant indicates edited alleles, upper right quadrant indicates wildtype alleles.

Figure S5

A

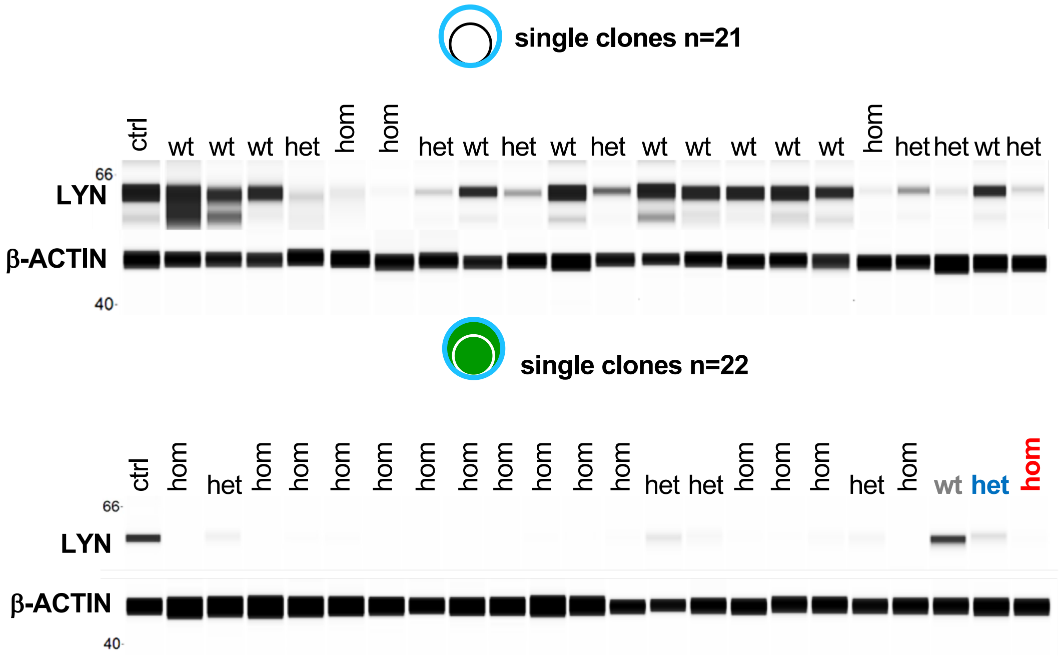

B

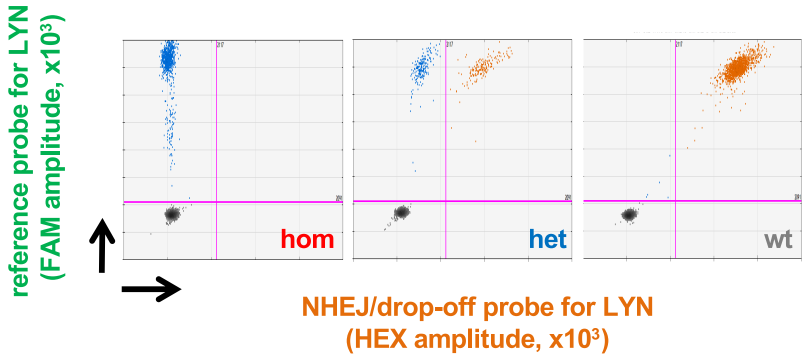

Figure S5 Analysis of the single cell clones (related to Figure 1E).

A) Capillary immunoassay**.** 21 GFP-negative and 22 GFP-positive single cell clones were analyzed for LYN protein expression by capillary protein immunoassay. “het” indicates protein reduction by around 50%, putatively indicating heterozygous gene-editing, while “hom” indicates protein reduction by > 80%, putatively indicating homozygous gene-editing; wt = wild type; ctrl = *GLuc* sgRNA targeting luciferase.

B) Verification of genotype by duplex probe PCR (ddPCR). From the GFP-positive single cell clones described in **A** (lower panel**)**, the genotype of one wildtype (wt, grey), one heterozygous (het, blue) and one homozygous (hom, red) gene-edited clone was verified by the ddPCR drop-off assay performed as described in Figure S3.

Figure S6

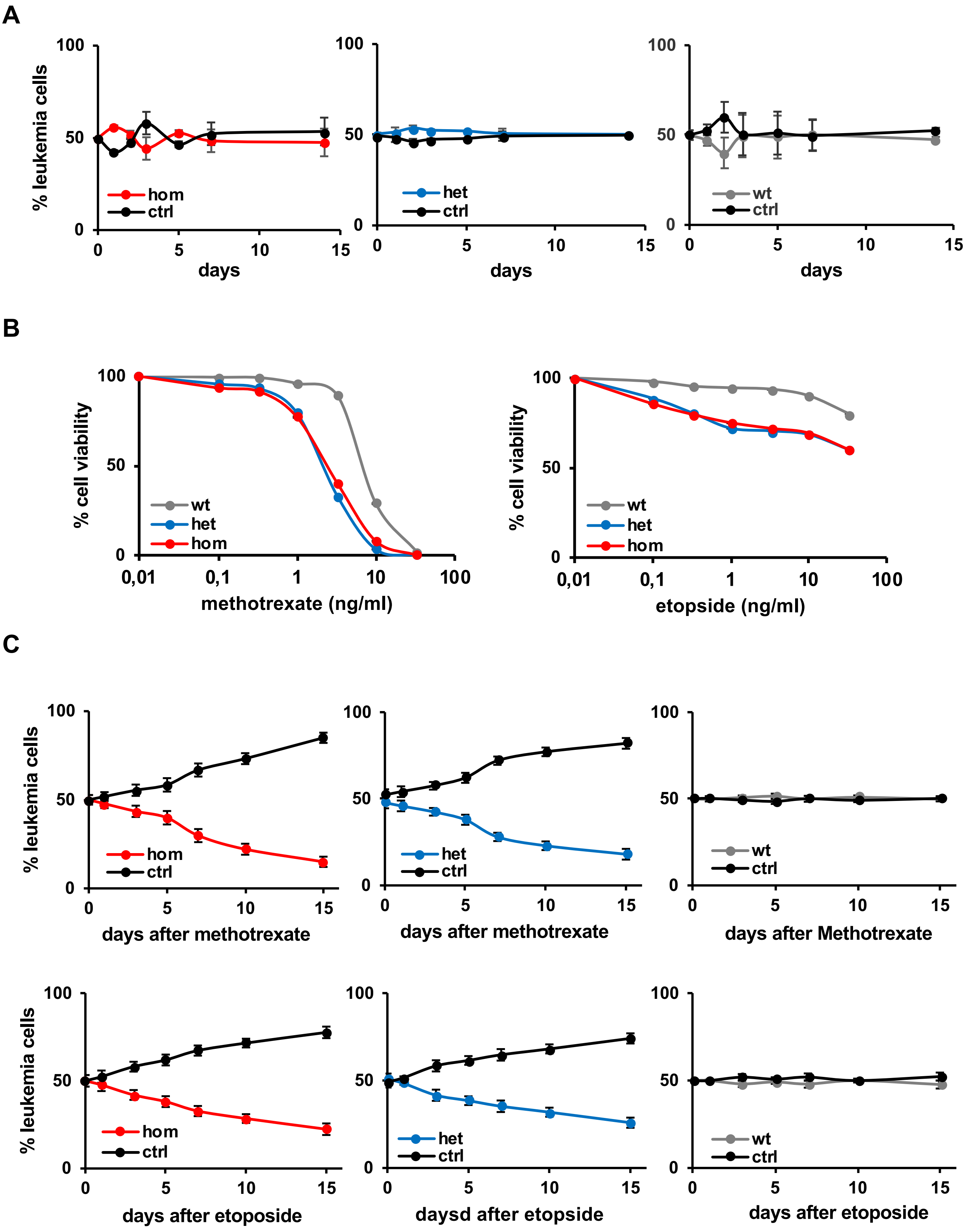

A
% leukemia cells
days
hom
ctrl
het
wt
B
% cell viability
methotrexate (ng/ml)
etopside (ng/ml)
wt
het
hom
C
% leukemia cells
days after methotrexate
days after Methotrexate
days after etoposide
daysd after etopside
hom
ctrl
het
wt

Figure S6 NALM-6 LYN KO single cell clones demonstrate increased sensitivity to chemotherapy

Growth and therapy response of the 3 GFP-positive single cell derived homozygous *LYN* KO (hom, red), heterozygous *LYN* KO (het, blue) and LYN wildtype (wt, grey) clones established as described in Figure S4 as compared to *GLuc* sgRNA expressing (GFP^+)-NALM-6 control cells (ctrl, black).

A) Competitive growth assay. Each of the 3 clones were mixed with untransduced NALM-6 control cells at a 1:1 ratio. Cell number was measured by FACS based on GFP expression at the indicated time points. Mean ± SD of 3 experiments is shown.

B) Dose response analysis to etoposide and methotrexate. Each of the 3 clones was treated with increasing concentrations of methotrexate (left) or etoposide (right) for 2 days before cell viability was measured by FACS based on FCS/SCC gating.

C) Heterozygous and homozygous loss of LYN equally sensitized cells to therapy. Each of the 3 clones was mixed with untransduced NALM-6 control cells at a 1:1 ratio and were treated with 1 ng/ml methotrexate or 10ng/ml etoposide for 15 days. Cell viability was measured by FACS based on FCS/SCC gating in the reporter positive and negative population at the indicated time points. Mean ± SD of 3 independent experiments is shown.

Figure S7

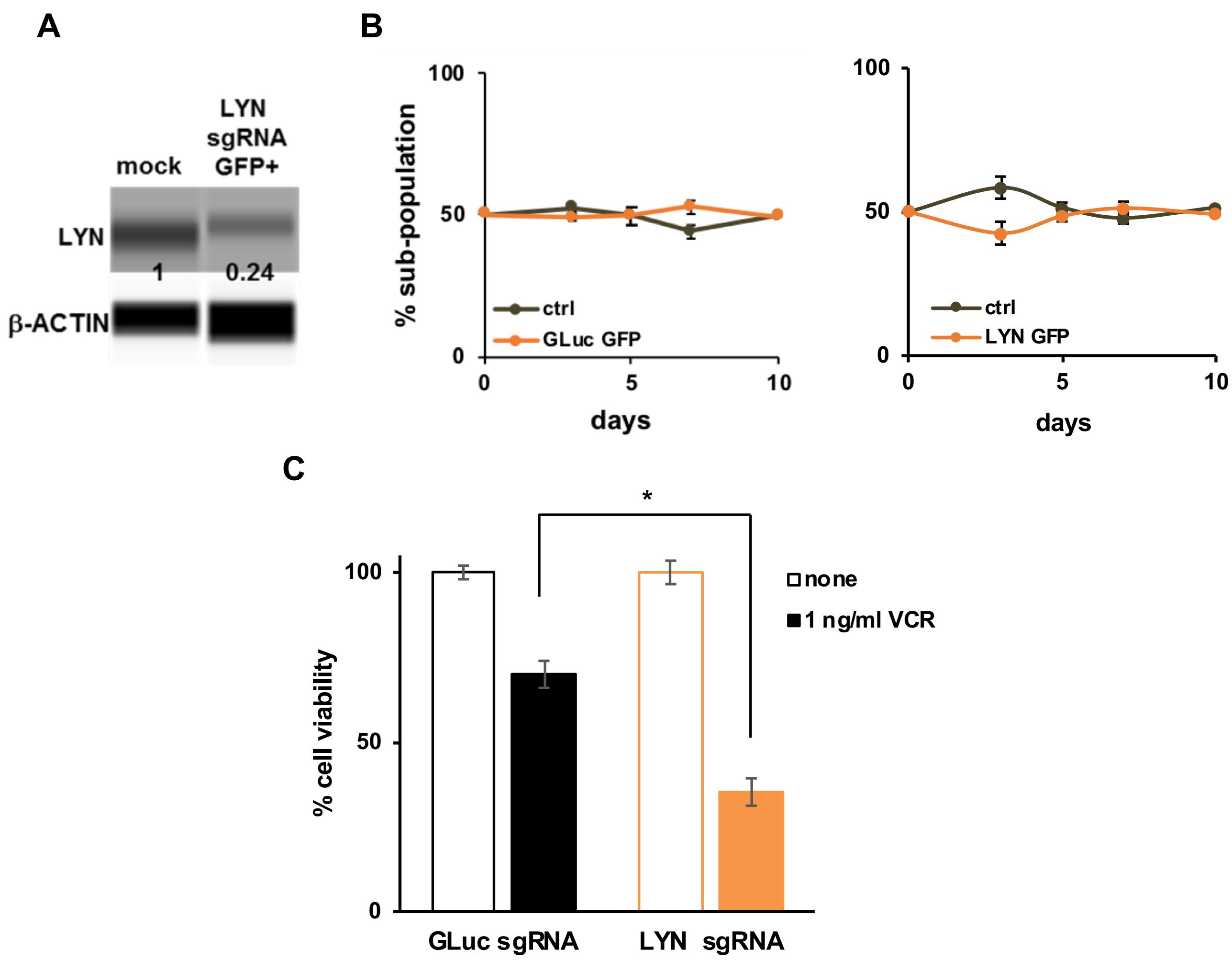

Figure S7 Enrichment of LYN KO cells using the reporter from the bulk population phenocopies effects observed in single cell clones.

NALM-6 cells expressing Cas9 and the reporter construct were transduced with *GLuc*- or *LYN*-sgRNA vectors. 5 days post transduction, sgRNA (mTaqBFP) and reporter (GFP) double-positive cells were sorted and subjected to further analysis.

A) <u>Capillary protein immunoassay.</u> LYN protein expression was analyzed in reporter-positive (GFP^+) population from *GLuc*- and *LYN* sgRNA-expressing cells, respectively. Values indicate fold change of normalized LYN protein expression relative to that of the *GLuc* sgRNA expressing control. .

B) <u>Competitive growth assay</u>. *GLuc*-ctrl KO (left) and *LYN* KO (right) cells were mixed at a 1:1 ratio with untransduced NALM-6 cells (ctrl) and percentage of cells in each subpopulation was measured by FACS based on GFP expression at the indicated time points. Mean ± SD of 3 independent experiments is shown.

C) CRIPSR/Cas9 LYN knockout cells increase chemosensitivity towards VCR. *GLuc*-ctrl KO and *LYN* KO cells were treated with 1 ng/ml VRC for 2 days. Cell viability was determined by FACS based on FCS/SSC gating. Mean ± SD of 3 independent experiments is shown. * $p < 0.05$.

Figure S8

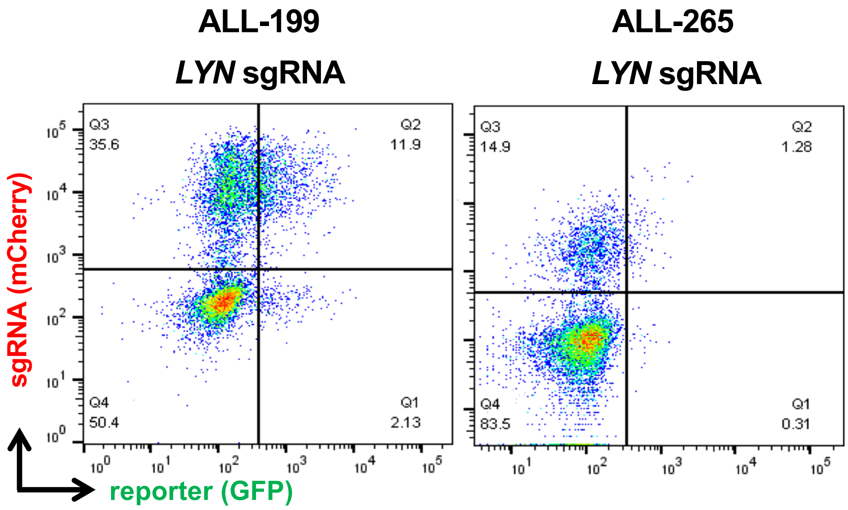

Figure S8 Generation of PDX KO cells.

Enrichment of reporter-positive PDX cells. Cas9-expressing PDX cells were lentivirally transduced with the *LYN*-sgRNA vector and the reporter constructs as described in **A**. After 5 days, cells were sorted according to GFP-expression and injected into mice.
